# Cumulative nitrogen enrichment alters the drivers of grassland overyielding

**DOI:** 10.1038/s42003-024-05999-9

**Published:** 2024-03-11

**Authors:** Miao He, Kathryn E. Barry, Merel B. Soons, Eric Allan, Seraina L. Cappelli, Dylan Craven, Jiří Doležal, Forest Isbell, Vojtěch Lanta, Jan Lepš, Maowei Liang, Norman Mason, Cecilia Palmborg, Noémie A. Pichon, Laíse da Silveira Pontes, Peter B. Reich, Christiane Roscher, Yann Hautier

**Affiliations:** 1 Ecology and Biodiversity group, Department of Biology, Utrecht University, Padualaan 8, 3584 CH Utrecht, The Netherlands; 2 Department of Ecology, Evolution, and Behavior, University of Minnesota, 1479 Gortner Ave, St Paul, MN 55108 USA; 3 Institute of Plant Sciences, University of Bern, Altenbergrain 21, 3013 Bern, Switzerland; 4 Centre for Development and Environment CDE, University of Bern, Mittelstrasse 43, 3012 Bern, Switzerland; 5 GEMA Center for Genomics, Ecology & Environment, Universidad Mayor, Camino La Pirámide, 5750 Huechuraba, Santiago Chile; 6 Data Observatory Foundation, ANID Technology Center No. DO210001, Eliodoro Yáñez 2990, 7510277 Providencia, Santiago Chile; 7 Department of Functional Ecology, Institute of Botany of the Czech Academy of Sciences, Zámek 1, 252 43 Průhonice, Czech Republic; 8 Department of Botany, Faculty of Science, University of South Bohemia, Na Zlaté stoce 1, 370 05 České Budějovice, Czech Republic; 9 Cedar Creek Ecosystem Science Reserve, University of Minnesota, 2660 Fawn Lake Dr NE, East Bethel, MN 55005 USA; 10 Landcare Research, Private Bag 3127, Hamilton, 3240 New Zealand; 11 Department of Crop production Ecology, Swedish University of Agricultural Sciences, 901 83 Umeå, Sweden; 12 Swiss Federal Research Institute WSL, Zürcherstrasse 111, CH-8903 Birmensdorf, Switzerland; 13 Rural Development Institute of Paraná – IAPAR-EMATER, Av. Euzébio de Queirós, s/n°, CP 129, CEP 84001-970, Ponta Grossa, PR Brazil; 14 Department of Forest Resources, University of Minnesota, 1479 Gortner Ave, St Paul, MN 55108 USA; 15 Institute for Global Change Biology, and School for the Environment and Sustainability, University of Michigan, 440 Church Street, Ann Arbor, MI 48109 USA; 16 UFZ, Helmholtz Centre for Environmental Research, Physiological Diversity, Permoserstrasse 15, 04318 Leipzig, Germany; 17 German Centre for Integrative Biodiversity Research (iDiv), Puschstrasse 4, 04103 Leipzig, Germany

**Keywords:** Biodiversity, Community ecology, Ecosystem services

## Abstract

Effects of plant diversity on grassland productivity, or overyielding, are found to be robust to nutrient enrichment. However, the impact of cumulative nitrogen (N) addition (total N added over time) on overyielding and its drivers are underexplored. Synthesizing data from 15 multi-year grassland biodiversity experiments with N addition, we found that N addition decreases complementarity effects and increases selection effects proportionately, resulting in no overall change in overyielding regardless of N addition rate. However, we observed a convex relationship between overyielding and cumulative N addition, driven by a shift from complementarity to selection effects. This shift suggests diminishing positive interactions and an increasing contribution of a few dominant species with increasing N accumulation. Recognizing the importance of cumulative N addition is vital for understanding its impacts on grassland overyielding, contributing essential insights for biodiversity conservation and ecosystem resilience in the face of increasing N deposition.

## Abstract (web summary)

15 multi-year grassland biodiversity experiments suggest that with increasing cumulative nitrogen addition, the effect of diversity on productivity becomes increasingly reliant on a small number of dominant species rather than on overall species richness.

## Introduction

Humans are enriching the environment with nitrogen (N) at an unprecedented rate^1^, and profoundly altering Earth’s ecosystems^2–4^. In grasslands, plant diversity^5,6^ and productivity^7,8^ change, as N accumulates over time^9,10^. Nitrogen enrichment, whether from experimental addition or atmospheric deposition, usually increases primary productivity by alleviating N limitation^8^. However, it reduces plant diversity by increasing competition for light^11–13^, acidifying soil^14–16^, reducing belowground niche dimensionality^17^, as well as accelerating the loss of rare species^5,18,19^ or even of common species^20^. Nitrogen addition may also alter the relationship between diversity and productivity^20–22^. If N addition weakens the positive effects of diversity on productivity^23,24^, this would have profound consequences for ecosystem management and our understanding of biodiversity-ecosystem functioning relationships. However, there is no consensus on how N affects biodiversity-ecosystem functioning relationships because the underlying mechanisms remain largely unexplored.

Nitrogen addition could alter how biodiversity affects productivity^20,25,26^. The effects of biodiversity on productivity can be quantified through net diversity effects, that is the extent to which species mixtures differ from the productivity expected from their constituent monocultures. Net diversity effects can be partitioned into two components: complementarity and selection effects^27^. Complementarity effects occur when species perform better in mixtures than expected from monocultures^28,29^. This can occur via several underlying mechanisms: 1) resource partitioning, where species exploit resources more completely in mixtures^30–32^; 2) greater facilitation in diverse mixtures^33–37^; or 3) reduced impacts of consumers, pathogens, or other natural enemies in mixtures^38,39^. Such mechanisms often operate more effectively in more diverse communities, leading to an increase in complementarity effects with species richness^21^. Nitrogen addition can decrease complementarity effects by decreasing positive interactions between legumes and other plants^32,35^, or by decreasing resource partitioning through a reduction in niche dimensionality and belowground nutrient trade-offs^31,40,41^. Alternatively, positive selection effects occur when species with a high productivity in monoculture increase their productivity in mixtures, while negative selection effects occur if the opposite happens. Nitrogen enrichment may enhance selection effects by increasing the dominance of some species and decreasing evenness, because alleviating N limitation may result in stronger competition for other resources, such as light or water^11,42,43^. Thus, N enrichment may either weaken or strengthen the effects of biodiversity on productivity, depending on whether it primarily affects complementarity or selection effects. Some empirical evidence suggests that N addition leads to a decrease in complementarity effects and an increase in the selection effects^26,32,44^.

**Fig. 1:**
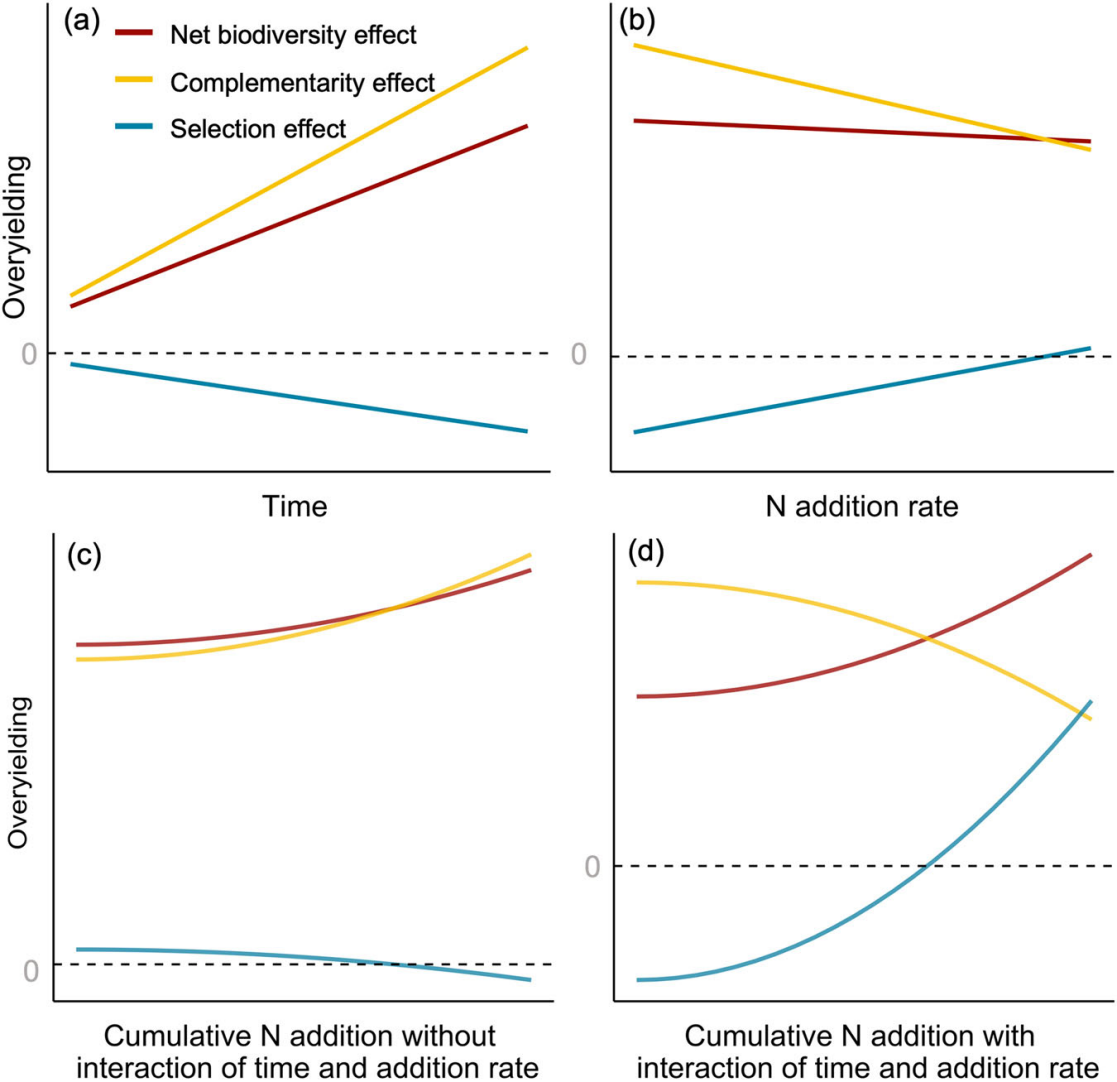
Conceptual figure about potential changes of overyielding and its components with cumulative nitrogen addition. Potential changes in net biodiversity effect, complementarity effect and selection effect with (**a**) time (without N addition; unit-year); (**b**) N addition rate (unit-kg/ha/year); (**c**) cumulative N addition (the total amount of N added across years; unit-kg/ha), when the effects of time and N addition rates are additive, these patterns without an interaction between the two effects were derived by multiplying the fitted trends in Fig. 1a, b; and (**d**) cumulative N addition, when the effects of time and N addition rates are multiplicative, these patterns with interaction between the two effects were derived by the fitted trend of multiplying data in Fig. 1a, b. Note that the x-axes are on the log scale for comparison with the results presented in this study.

The effects of N addition on complementarity and selection effects may also change over time. Complementarity effects typically increase over time, leading to increased overyielding as plant communities mature, while selection effects decrease^45–47^ (Fig. 1a). Under N addition, complementarity effects may decline linearly, while selection effects may increase linearly with N addition (Fig. 1b). If these two effects (Fig. 1a, b) are additive, then the combination of an increase with time and a decrease with N addition rate will result in a convex relationship between cumulative N addition and net biodiversity effects (Fig. 1c). This pattern may be driven by a convex relationship between cumulative N addition and complementarity effects, which is partly counteracted by a concave relationship between cumulative N addition and selection effects. In this case, nutrient enrichment would gradually erode the positive effects of biodiversity on ecosystem functioning. This erosion would likely occur even when nutrient enrichment increases the strength of selection effects, if selection effects remain a small fraction of the total effects of biodiversity on productivity. However, it is also possible that N addition interacts with time, leading to multiplicative effects of cumulative N addition. An interaction would occur if effects of N addition on complementarity and selection effects strengthen over time. For example, increasing N enrichment could cause a decrease in species richness due to the recycling of N through litter^48^ and soil acidification^6^, thereby preventing the increase in complementarity effects over time^49^. Alternatively, selection effects could increase more over time^44^ due to gradual changes in the soil microbial community and abiotic environment^50^. Selection effects may therefore contribute a larger fraction of biodiversity effects than would be the case without long term N addition (Fig. 1d). Overall, the impacts of nitrogen enrichment on the relationship between plant diversity and productivity can be complex, and its effects may vary depending on the N addition rate and the duration of nitrogen addition. To our knowledge, no previous study has quantified the impacts of cumulative nitrogen addition on overyielding and its underlying processes, nor has any experiment explored the interaction of N addition rate and duration in a full factorial design. This research gap results in an incomplete understanding of the effects of N addition on net biodiversity effects over time. Understanding these relationships is crucial for predicting how long-term eutrophication may alter the effects of biodiversity on ecosystem functioning in the future.

Here, we use a meta-level synthesis to determine the main effects of plant species richness and N addition on productivity, using multi-year experiments that manipulated both factors (Supplementary Table S1). We evaluate the impacts of N addition rate, time and cumulative N addition (over time), on net biodiversity, complementarity and selection effects. Our hypotheses are (see Supplementary Table S2 for fully detailed hypotheses and mechanisms):

H1 Nitrogen addition:

H1a: Nitrogen addition treatment (binary): complementarity effects decrease, and selection effects increase with N addition, resulting in no overall change in net biodiversity effects. Effects of N addition on complementarity and selection effects are more pronounced at higher species richness.

H1b: Nitrogen addition rates: Effects of N addition on complementarity and selection effects are more pronounced at higher rates of N addition (Fig. 1b).

H2 Nitrogen addition treatment * Time: under ambient conditions, complementarity effects increase, and selection effects decrease with time (year). We expect larger increases in complementarity effects than decreases in selection effects, leading to increase in net biodiversity effects over time (Fig. 1a). The effects of N addition counteract the effects of time on complementarity effects and selection effects.

H3 Cumulative N addition: Additive effects between N addition rate and time (year) will lead to a convex relationship of complementarity effects and a concave relationship of selection effects with increasing cumulative N addition (Fig. 1c). If there is an interaction between N addition rate and time (year), complementarity effects may decrease and selection effects increase more rapidly at higher levels of cumulative N addition. The shift from complementarity to selection effects will lead to a convex relationship between net biodiversity effects and cumulative N addition (Fig. 1d).

## Results

### Impacts of the nitrogen addition treatment

**Fig. 2:**
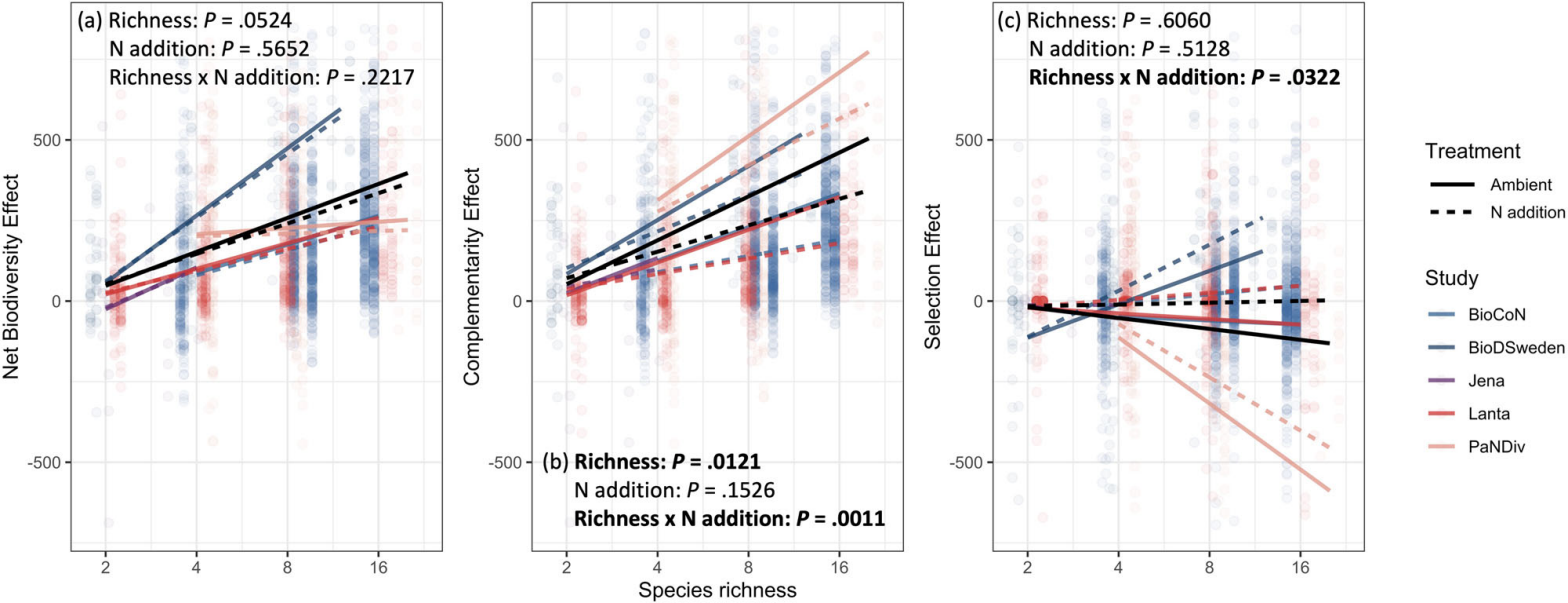
The impacts of nitrogen addition treatment on the relationships between species richness and overyielding. The impacts of N addition on the relationships of species richness with net biodiversity effects (**a**), complementarity effects (**b**) and selection effects (**c**). Black lines indicate fixed effects, and colored lines indicate random effects. Note that the x-axes are on the log-2 scale, the y-axes are on the original scale with unit g/m^2^/year.

We found a marginally significant increase in net biodiversity effects with species richness (Fig. 2a; Supplementary Table S3). This increase was largely due to an increase in complementarity effects with species richness, as there was no significant change in selection effects. Nitrogen addition interacted with species richness and strongly reduced the complementarity effects (Fig. 2b) and reduced the negative selection effects (Fig. 2c) at high species richness. Despite these changes in complementarity and selection effects, the relationship between species richness and net biodiversity effects remained unchanged by N addition, because the opposing effects of nitrogen addition on complementarity and selection effects canceled each other out.

### Impacts of nitrogen addition rate

**Fig. 3:**
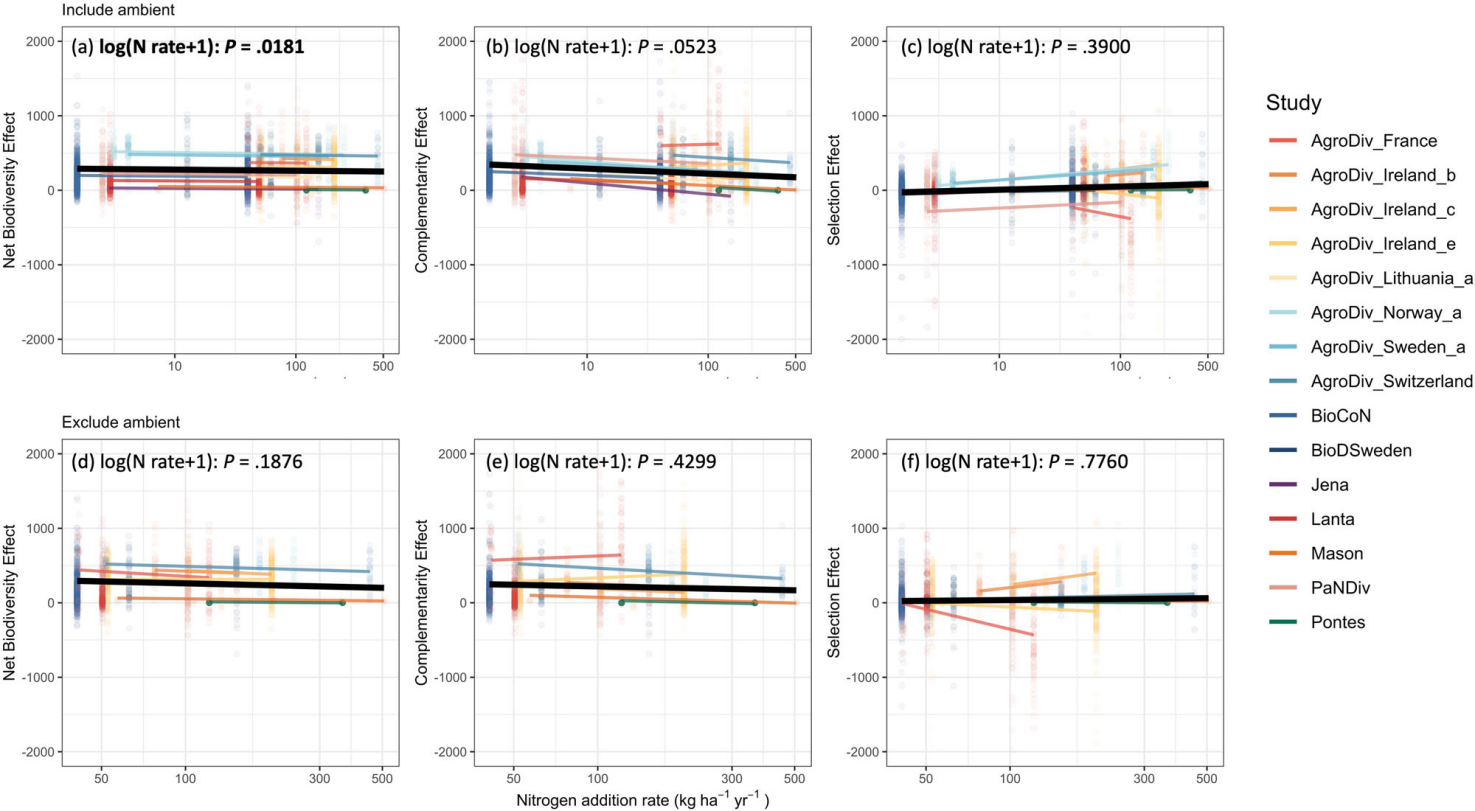
The impacts of nitrogen addition rate on overyielding. The impacts of N addition rate (from both experimental addition and atmospheric deposition) on net biodiversity effects (**a**, **d**), complementarity effects (**b**, **e**) and selection effects (**c**, **f**) including (**a**–**c**) or excluding (**d**–**f**) the ambient plots. Black lines indicate fixed effect, colored lines indicate random effect based on individual study. The X-axes are on the log scale with unit kg/ha/year, the y-axes are on the original scale with unit g/m^2^/year.

We found that higher N addition rates marginally reduced complementarity effects but did not affect selection effects (Fig. 3b, c; Supplementary Table S3), leading to reduced net biodiversity effects with increasing N addition rate (Fig. 3a). However, these relationships were mainly driven by the difference between ambient and fertilized plots (with experimental N addition), i.e., the significant and marginally significant relationships with ambient and fertilized plots became non-significant when ambient plots were removed (Fig. 3d, e; Supplementary Table S3). As a result, experimental N addition decreased complementarity effects (Supplementary Fig. S1b) but increased selection effects (Supplementary Fig. S1c), regardless of the annual rate of nutrient enrichment. These opposite but proportional responses led to no effect of experimental N addition on the net biodiversity effects (Supplementary Fig. S1a).

### Impacts of nitrogen addition over time

By analyzing the long-term BioCoN experiment we found that N addition treatment interacted with time, influencing both complementarity effects and selection effects (Supplementary Fig. S2). Specifically, N addition reduced the increase of complementarity effects with time (Supplementary Fig. S2b) and offset the decrease in selection effects with time (Supplementary Fig. S2c). These opposing effects of time and nitrogen addition led to decreasing net biodiversity effects over time (Supplementary Fig. S2a).

### Impacts of cumulative nitrogen addition

**Fig. 4:**
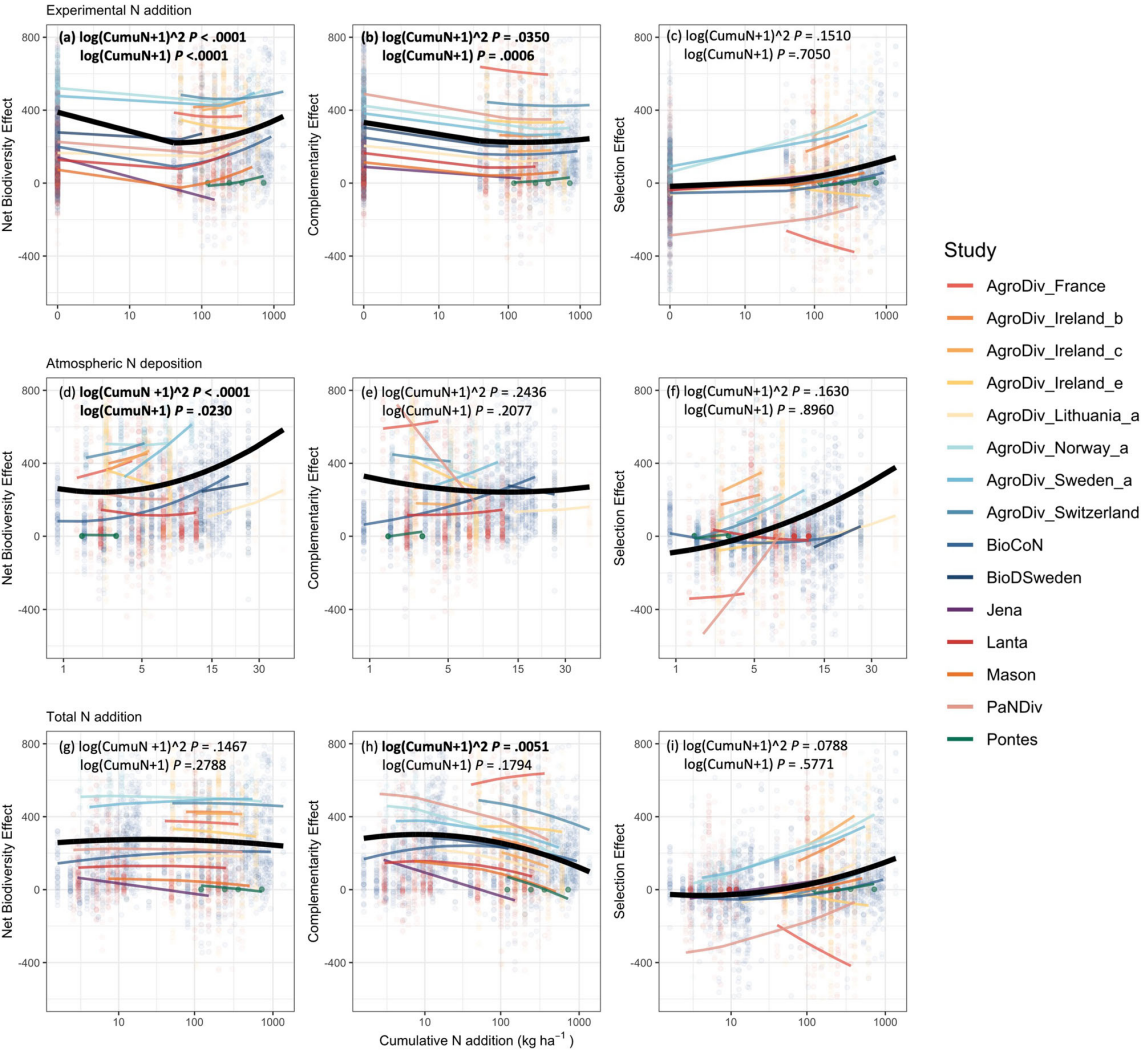
The impacts of cumulative nitrogen addition on overyielding. The impacts of cumulative N addition on net biodiversity effects (**a**, **d**, **g**), complementarity effects (**b**, **e**, **h**) and selection effects (**c**, **f**, **i**). Cumulative N addition includes inputs from experimental addition (**a**–**c**), atmospheric deposition (**d**–**f**) and both (**g**–**i**). Black lines indicate fixed effect, colored lines indicate random effect. The x-axes are on the log scale with unit kg/ha, the y-axes are on the original scale with unit g/m^2^/year.

Across all experiments, we found a convex relationship between cumulative experimental N addition and net biodiversity effects (Fig. 4a; Supplementary Table S3). Complementarity effects decreased first and then levelled off with increasing cumulative experimental N addition (Fig. 4b), while selection effects increased continuously (Fig. 4c). As expected, similar trends were found with atmospheric N deposition (Fig. 4d–f). However, when combining inputs from both experimental N addition and atmospheric N deposition, complementarity effects decreased more rapidly (Fig. 4h), while selection effects increased more rapidly (Fig. 4i) at higher levels of cumulative N addition. These counteracting effects were proportional, leading to a non-significant relationship between net biodiversity effects and total amount of cumulative N addition from both experimental addition and atmospheric deposition (Fig. 4g).

## Discussion

Our study reveals that time and N addition rate interactively affect overyielding and its drivers. Low levels of cumulative experimental N addition decrease net biodiversity effects and complementarity effects, while high levels of cumulative N addition increase net biodiversity effects and selection effects. This finding highlights that cumulative N addition alters overyielding by modulating the relative contributions of complementary and selection effects.

Our synthesis of 15 grassland experiments is consistent with previous findings that overyielding is robust to nutrient enrichment^21^. However, our results indicate that this lack of effect occurs due to contrasting effects on the different components of net biodiversity effects, with a decrease of complementarity effects and a proportional increase in selection effects with nutrient addition^32^ (Fig. 2). Nitrogen addition reduces complementarity effects more strongly at higher levels of species richness, potentially due to changes in underlying ecological mechanisms. One potential explanation is that with increasing N, plant species may facilitate each other less^29^. Weaker facilitation may be partially attributes to a lower abundance or lower N_2_ fixation rate of legumes and, therefore, reducing N fixation^36,51–53^. However, this is likely not the only explanation for the observed decrease in complementarity effects, as our dataset includes experiments without legumes^54^ (PaNDiv experiment). Another potential explanation is that N addition may modify the community of beneficial belowground mycorrhizal fungi or rhizobacteria, thereby reducing positive interactions mediated by microbes^55–57^. Moreover, N enrichment may cause the loss of plant species by alleviating N limitation and promoting interspecific competition^11,13,58,59^. These effects are especially pronounced in species-rich communities^16^, as the increased resources reduce the opportunity for different species to partition resource utilization in space, time, or form, leading to larger decreases in plant species richness, complementarity effects and thereby productivity in diverse communities^20^.

We also find that N addition decreases the negative effect of species richness on selection effects. This finding is in contrast with previous studies reporting non-significant interactions between N and richness^21,60^. This difference may be explained by the fact that our analysis includes studies that added relatively high amounts of N (e.g., 500 kg/ha/year^32^ or 450 kg/ha/year^61^), cover larger species richness gradients (e.g., for 1–20 species in PaNDiv experiment^54^), and use an agricultural-based species pool instead of a broader species pool^61^. The larger increase in selection effects with N addition at higher diversity (Fig. 2c) may occur because species mixtures with higher diversity have a greater chance of including species that are more sensitive to the change in N availability. By growing faster and taller, these species are able to capture more light and shade the other species, leading to an increased competitive ability and, therefore, increased selection effects with N addition^62–65^.

Our study reveals that the responses of net biodiversity, complementarity and selection effects to N enrichment are independent of the annual rate of experimental N addition (Fig. 3d–f). The lack of effect of N addition rate on overyielding could be due to the complementary utilization of N by plants with different functional traits, which maintains ecosystem productivity along a N addition rate gradient^66^. However, it is important to note that any threshold of annual N addition rate may be too low to be detected with the addition rates used in current experiments (Fig. 3; Supplementary Table S3). In addition, a large proportion of the impact of different N addition rates depends on variation of annual N addition rates among experiments; other factors that vary among the studies could also obscure the effects of N addition rate, including the form of N added, soil type, and how much N was initially available at the site^67^ (Supplementary Table S1). More experiments fully crossing diversity with multiple levels of N addition would be needed to fully test this idea.

Overyielding may be regulated more by cumulative N addition over time than by the annual rate of N added. In our data, some studies apply a relatively low amount of N annually for a relatively long period of time (e.g., 40 kg/ha/year for 23 years)^20,68,69^, while others apply a higher amount of N annually for a shorter period of time (e.g., 360 kg/ha/year for 3 years)^70^. Confirming previous results^46,47^, we find that complementarity and selection effects changed over 23 years in BioCoN (Supplementary Fig. S2). Nitrogen addition also interacts with time to affect overyielding and reduce the increase of complementarity effects with time, while shifting selection effects from negative to positive over time (Supplementary Fig. S2b, c). Considering cumulative N addition over time, we find a faster decrease in complementarity effects at low levels of cumulative N addition (Fig. 4b, e). This may indicate a higher sensitivity of biotic interactions to low levels of cumulative N addition. Our results suggest that net biodiversity effects may level off or even bounce back in the long run under cumulative N addition, due to increases in overyielding following increased species dominance^4,45^. However, this may result in the community behaving as a functional monoculture despite a positive net biodiversity effect. Specifically, the consistent increase in selection effects with cumulative N addition may overwhelm the decrease in complementarity effects, resulting in a convex relationship between net biodiversity effects and cumulative N addition (from either experimental addition or atmospheric deposition; Figs. 1d and 4a, d). This result contrasts with the expected concave relationship based on the null hypothesis that the effects of N addition rate and time are independent (Fig. 1c). Instead, this convex relationship suggests a strong interaction between the impact of N addition rate and the impact of time, further indicating a shift in the relative importance of biodiversity effects, from complementarity to selection effects, under N addition. This shift in relative importance occurs regardless of the pathway of N addition, i.e., atmospheric N deposition or fertilization. Similar convex relationships between net biodiversity effects and cumulative N addition are found from experimental addition (at higher levels, generally 40–400 kg/ha/year) or atmospheric deposition (at lower levels, generally 0–40 kg/ha/year) alone. These two convex relationships may result in a non-significant relationship between net biodiversity effects and cumulative N addition when combining both pathways of N inputs.

The shift in the relative importance of complementarity and selection effects under increasing cumulative N addition may be due to changes in community structure, i.e., changes in evenness. That is, the reduction of N-limitation over extended periods may favor large and fast-growing species, leading to an increase in selection effects^36,71,72^. Additionally, N addition may lead to asymmetric competition for light in mixtures. This increased asymmetric competition may simultaneously reduce opportunities for species complementarity in resource use and intensify the effects of competitive hierarchies on species relative abundances^47,65,73^. Furthermore, communities dominated by highly productive species are usually more susceptible to climate change, leading to higher variation through time^3,74^. However, our analysis does not account for potential indirect effects of N addition through species composition on overyielding^75,76^. We also note that our estimates of cumulative N addition do not account for N losses due to N leaching or biomass removal. Some experiments included in the present study, e.g., the Jena Experiment and the PaNDiv experiment, remove all aboveground biomass annually, while others do not, e.g., BioCON. Removal of biomass and the associated nitrogen may lead to a decrease in soil N that is accessible to plants over time, which would have otherwise been recycled within the system^48,77,78^. Additionally, biodiversity effects on soil N mineralization rates also have been found to shift from negative to positive over time, indicating that species rich communities could have higher N retention than species poor communities with increasing N addition duration^79^. The interaction between N addition and species richness found in our study implies that future studies should consider the cumulative amount of N when assessing the impacts of N addition on overyielding. This involves incorporating N rates and duration in a full factorial design and measuring N content in litter, in removed biomass, or within the plant-soil system.

To sum up, our study reveals that cumulative N addition influences the ecological mechanisms underlying overyielding, thereby expanding our understanding of how global change affects biodiversity-ecosystem functioning relationships across grasslands. Specifically, with increased cumulative N addition, we observe a shift in the relative importance of the components of net biodiversity effects from complementarity to selection effects. While cumulative N addition boosts selection effects, it does not generate a net impact on overyielding due to the diminishing role of complementarity effects in high diversity communities. Our results suggest that the effect of biodiversity on productivity becomes increasingly reliant on a small number of dominant species rather than on overall species richness^38^, thereby amplifying ecosystem susceptibility to environmental fluctuations associated with global change, such as disease outbreaks^80–82^, climate variability^74,83,84^, and disturbance^65^.

## Methods

### Data collection

We conducted a meta-level synthesis to explore the impact of N addition on overyielding in grassland ecosystems. We had three requirements for datasets to be included in this study: 1) the experiments needed to cross a gradient of sown plant species richness with a N addition treatment; 2) the experiments needed to measure species-level biomass (g/m^2^) at the plot scale for each plant community, including monocultures; 3) biomass should be measured at earliest in the second year after the establishment of experiments establishment. For studies that collected biomass in the same location more than once a year (No. 4, 5, 7–15 in Supplementary Table S1), we summed biomass from multiple harvests per year as a proxy for aboveground annual productivity (g/m^2^/year), to enable comparison across studies. In total, 15 grassland studies met our criteria, with observations from 1504 plots. The selected studies were distributed across ten countries, with the richness of sown species ranging from 1 to 20; the number of years for which we had biomass data ranging from 1 to 23 years; and N addition rate ranging from 0 to 500 kg/ha/year.

We then tested our hypotheses using the studies meeting our selection criteria (Supplementary Table S1). To test the impacts of N addition as a binary factor (H1a), we used studies that included both unmanipulated ambient plots and N addition plots (studies No. 1–5, 7, 12–14); to test whether overyielding varied with species richness under N addition, we used studies with more than two species richness levels, in addition to monocultures (studies No. 1–3, 5, 7); H2); to test the interaction between the effects of N addition (binary) and the effects of time (H2), data from the plots with N but not CO_2_ enrichment at BioCoN experiment (study No. 1) was used since it has run continuously for 23 years, while other studies lasted less than 5 years (studies No. 2–15); and to test the effects of N addition rate (H1b) and cumulative N addition (H3) on overyielding, the full dataset was used (studies No. 1–15).

### Diversity effects calculation

Relative yield of species i $(R{Y}_{i})$ and the total relative yield of the mixture $({RYT})$ were calculated as in Harper (1977)^85^:

$$R{Y}_{i}={Y}_{i}/{M}_{i}$$

$${RYT}=\sum R{Y}_{i}$$

where ${Yi}$ and ${Mi}$ are the observed yield of species i in mixture and monoculture, respectively.

The change in the relative yield $(\Delta {RY})$, net biodiversity effect, complementarity effect and selection effect were calculated as in Loreau and Hector^27^:

$$\Delta {RY}=R{Y}_{i}-R{Y}_{e,i}$$

$${Net}\,{biodiversity}\,{effect}=\Sigma {Y}_{i}-\Sigma \left(R{Y}_{e,i}\times {M}_{i}\right)$$

$${Complementarity}\,{effect}=n\times \bar{M}\times {\overline{\Delta RY}}$$

$${Selection}\,{effect}=n\times {{{{{\mathrm{cov}}}}}}\left({\bar{M}},\Delta {RY}\right)$$

where $R{Y}_{e,i}$ is the sown proportion of species i, $\bar{M}$ is the mean above-ground productivity (g/m^2^/year) in a monoculture of each sown species and *n* is sown species richness. Note that we added 1 (the 1.25% left tail of distribution in our full dataset) to all the monoculture yields in our analysis, since relative yield approaches infinity with small monoculture yield. Complementarity and selection effects were calculated via the *partitionBEFsp* package with corrected covariance^86^.

### Statistics and reproducibility

We fitted separate mixed effect models to assess the effect of N addition on net biodiversity, complementarity, and selection effects. We partitioned the net biodiversity, complementarity, and selection effects following Loreau and Hector^27^, to capture both overyielding and underyielding. For the general impact of N addition treatment (H1a), we included the N addition treatment, experimental site (represented by different studies), and their interaction as fixed effects, and study specific plot ID nested in year as random effects. We also accounted for repeated measurements on the same plot via a first-order autoregressive temporal autocorrelation structure. After fitting the model, we calculated the estimated mean response under ambient or N addition treatments using the *emmeans* package^87^. We also tested how biodiversity effects changed with species richness under N addition by including N addition treatment (0, ambient; 1, N addition), sown species richness and their interaction as fixed effects, and species richness nested in study ID as a random effect. To be consistent with the design of the diversity gradients, we used log2-transformed sown species richness to represent species richness. To explore the impacts of N addition rate (H1b), we included N addition rate as a fixed effect and allowed random intercepts and slopes among different studies. To better meet the assumptions of our model, we used log-transformed N addition amount per year to represent annual N addition rate. To disentangle the impacts of N addition treatment (binary) and the impacts of N addition rate, we explored two conditions: including plots both with and without N experimental addition (Include ambient), or only plots with N addition (Exclude ambient). To represent the actual N addition per plot (including on control plots) and to account for spatial variation of N deposition rate, the annual N addition rate was the sum of N addition from experimental addition and atmospheric deposition. The total atmospheric deposition (NO^−^ + NH^+^, both wet and dry) rate was extracted according to the location of each study site^88,89^. A static deposition rate was used here to cover spatial variation of deposition, not its temporal variation.

To explore the interaction between impacts of N addition treatment and the impacts of time (H2), we included N addition treatment, year and their interaction as fixed effects, and species richness as a random effect.

To explore the effect of cumulative N addition (H3), we multiplied N addition (experimental addition + atmospheric deposition, in kg/ha/year) by the number of years over which inputs occurred. We also compared models with cumulative N addition to those with an interaction of time and N addition rate, and models with cumulative N addition performed better based on Akaike information criterion (Supplementary Table S4). Based on our hypothesis of an interaction between the effects of time and nitrogen addition rates, we added a second order polynomial term for the impacts of cumulative N addition and assessed its goodness of fit based on the Akaike information criterion (Supplementary Table S5). The effects of evenness change with cumulative nitrogen addition and time was tested (Supplementary Table S6). Based on the goodness of fit, we set both the first and second order terms of cumulative N addition as fixed effects and allowed random intercepts and slopes among different studies (see Supplementary Table S3 for fully detailed model settings). In addition, we explored whether the impacts of cumulative N addition were regulated by different inputs: only experimental addition, only atmospheric deposition or both combined, due to the cascading effects of N from these inputs at different rates. All of the analyses were conducted using R version 4.0.5^90^, within RStudio IDE^91^. The following packages were used: AICcmodavg^92^, dplyr^93^, emmeans^87^, itsadug^94^, lme4^95^, lmerTest^96^, magrittr^97^, MuMIn^98^, nlme^99^, optimx^100,101^.

### Reporting summary

Further information on research design is available in the Nature Portfolio Reporting Summary linked to this article.

### Supplementary information

Supplementary metarials

Reporting Summary

## Data Availability

The original data sets used in this data synthesis are available from data repositories of included studies, or upon request to data owners. The detailed information of included studies was documented on Table S1.

## Code availability

All code used in this study available at the figshare repository with: 10.6084/m9.figshare.25153151.
