# Supplementary material for: Cumulative nitrogen enrichment alters the drivers of grassland overyielding
Source: Commun Biol. 2024 Mar 11;7:309. doi: 10.1038/s42003-024-05999-9 (PMC10928195; doi:10.1038/s42003-024-05999-9)
Supplement: Supplementary file 1 — Supplementary metarials [file 42003_2024_5999_MOESM1_ESM.pdf]

Supporting Information

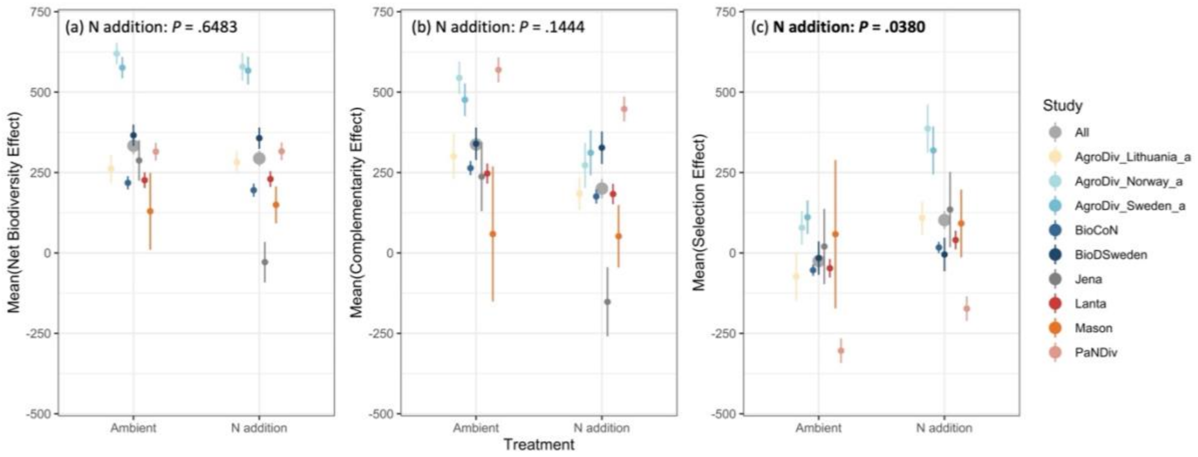

Figure S1. N addition treatment The impact of experimental N addition on the net biodiversity effects (a), the complementarity effects (b) and the selection effects (c) across (grey points) or on each individual study (colored points). The y-axes are on the original scale with unit g/m^2/year.

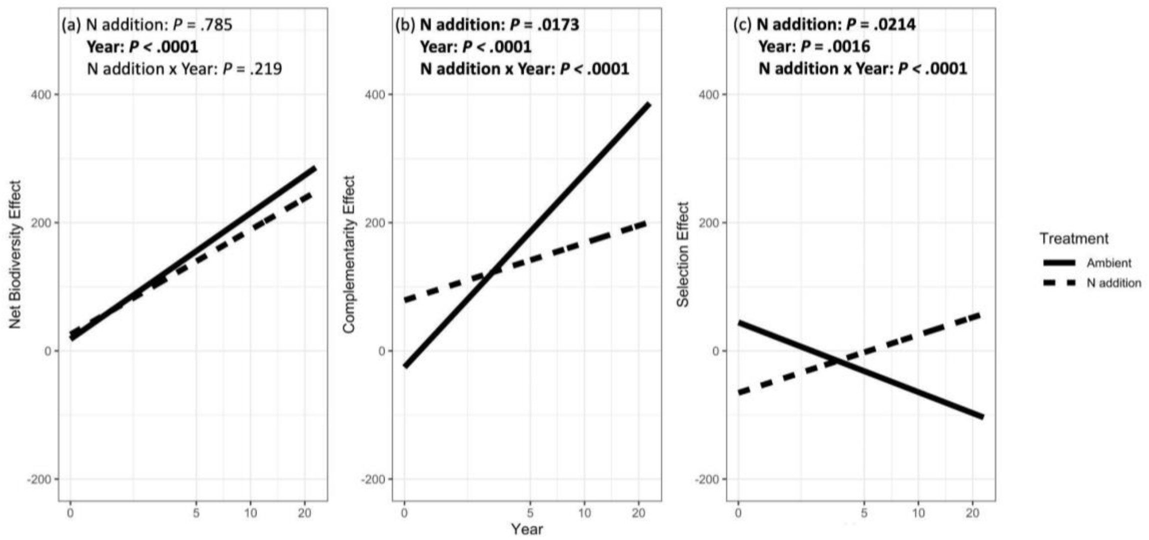

Figure S2. Year x N addition treatment The impact of N addition and its duration (year) on net biodiversity effects (a), complementarity effects (b) and selection effects (c) for BioCoN experiments (23 years). The BioCoN experiment was used to represent the temporal trend (Fig. 1) because it is the only experiment in our study that has more than 5 years of data. The x-axes are on the log scale with unit year, the y-axes are on the original scale with unit g/m^2/year.

Table S1. Overview of dataset

	Experiment	Region	Study	Data repositories or owners' contact	Treatment	Fertilizer type	Nitrogen added amount (kg.ha-1.y-1)	Nitrogen atmospheric deposition (kg.ha-1.y-1)	Period	Number of species	Used in
1	BioCoN	Cedar Creek, Minnesota, USA	Isbell et al., 2013; Reich et al., 2001	https://portal.edirepository.org/nis/mapbrowse?packageid=knb-lter-cdr.302.newest	AmbientN,N	NH4NO3	0,40	0.8412	1998-2020	1,4,9,16	Fig. 2-4, S1, S2
2	BioDSweden	Umea, Sweden	Palmborg, unpublished	Cecilia Palmborg (cecilia.palmborg@slu.se)	AmbientN,N	NH4NO3	0,50	12.8280	2002-2003	1,2,4,8,12	Fig. 2-4, S2
3	Lanta	Benesov/Lipou, Czech Republic	Lanta & Leps 2007	Vojtech Lanta (vlanta@centrum.cz); Jan Lepš (suspa@prf.jcu.cz)	AmbientNPK, NPK	Liquid fertilizer (Synferta P-NPK, AGRO CS Ceska´Skalice, CZ)	0, 48N (400 kg/ha NPK with 12%N+12%P+12%K)	2.3688	2002-2006	1,2,4,8,16	Fig. 2-4, S2
4	Mason	the North Island, New Zealand	Mason et al., 2020	Norman Mason (masonn@landcareresearch.co.nz)	AmbientN,N	Urea	0,50,100,200,350,500	7.0888	2015.06-2016.05	1,5	Fig. 3-4, S2

5	PaNDiv	Münchenbuchsee, Switzerland	Pichon et al., 2020	Eric Allan (eric.allan@unibe.ch); Seraina Cappelli (scappell@umn.edu); Noémie Pichon (noemie.pichon@wsl.ch)	AmbientN,N	Urea	0,100	1.9312	2017-2020	1,4,8,20	Fig. 2-4, S2
6	Pontes	Theix, France	Pontes et al., 2012	Laise da Silveira Pontes (laisepontes@hotmail.com)	N	NH4NO3	120,360	1.5924	2003-2004	1,6	Fig. 3-4
7	Jena_Roscher	Jena,Germany	Roscher et al., 2016	Christiane Roscher (christiane.roscher@ufz.de)	AmbientNPK, NPK	N, P, K	0,150(N)+65.4(P)+124.5(K)	2.2940	2009	1,2,3,4	Fig. 2-4, S2
8		France			N	NH4NO3	40,120	1.4128	2004-2006	1,4	Fig. 3-4
9		Ireland_b			N	NH4NO3	75,150	2.7513	2006-2007	1,4	Fig. 3-4
10		Ireland_c			N	NH4NO3	100,200	2.8227	2006-2007	1,4	Fig. 3-4
11	AgroDiv	Ireland_e	Kirwan, 2014 Metadata	https://www.esapubs.org/archive/ecol/E095/232/	N	NH4NO3	50,200	2.6253	2007-2009	1,2,4	Fig. 3-4
12		Lithuania_a			AmbientN,N	NH4NO3	0,120	14.1593	2003-2005	1,4	Fig. 3-4, S2
13		Norway			AmbientN,N	NH4NO3	0,240	2.6045	2004-2006	1,4	Fig. 3-4, S2
14		Sweden_a			AmbientN,N	NH4NO3	0,180	3.6901	2003-2005	1,4	Fig. 3-4, S2

15	Switzerland	N	NH4NO3	50,150,450	1.7409	2003-2005	1,4	Fig. 3-4

*Ireland_b, Ireland_c and Ireland_e represents three different experimental sites in Ireland.

Table S2. Hypotheses and mechanisms on overyielding and how it changes through time, nitrogen addition, and cumulative nitrogen addition.

	Relationship	Slope	Hypotheses and mechanisms	Reference
Overyielding				
	Species richness -> Complementarity effects	Positive	Communities with more species have higher potential to partition resource niche or facilitate each other, thus leading to a higher complementarity effect	Kahman et al., (2006); Wright et al., (2017)
	Species richness -> Selection effects	Negative or neutral	The promotion of the species in mixture that performed poorly in monoculture leads to a decrease of selection effects with higher number of species	Jiang et al., (2008)
	Species richness -> Net biodiversity effects	Positive	The positive relationship between biodiversity and complementarity effects contributes more than the negative relationship between biodiversity and selection effects, leading to a positive relationship between biodiversity and the net biodiversity effects	Craven et al., (2016)
H1a: Overyielding change under nitrogen (N) addition				
	N addition -> Complementarity effects	Negative	N addition reduces complementarity effects by reducing niche complementarity or facilitation, leading to a weaker relationship between richness and complementarity effects compared to ambient conditions, and the response is larger with higher species richness.	Craven et al., (2016)
	N addition -> Selection effects	Positive	N addition increases selection effects by increasing the dominance of nitrophilous species and their competition with other species, leading to a more positive relationship between richness and selection effects compared to ambient conditions, and the response is larger with higher species richness	Kirwan et al., (2007)
	N addition -> Net biodiversity effects	TBD	A decrease in complementarity effects that is proportional with the increase of selection effects leads to a robust relationship between the net biodiversity effects and diversity under N addition	NA
H1b: Overyielding change under increasing N addition rate				
	N addition rate -> Complementarity effects	Negative	The increase of N addition rate could bring larger decrease to complementarity effects due to larger variation in plant species composition and soil biota under higher annual N addition rate	Song et al., (2011); You et al., (2017)

N addition rate -> Selection effects	Positive	The increase of N addition rate could increase selection effects by enhancing the dominance of certain species. Low annual N addition rate might promote fast-growing exploitative species; while slow-growing conservative species that produce more leaf dry matter tend to prevail under higher N addition rate instead.	Wardle et al., (2004); Orwin et al., (2021)
N addition rate -> Net biodiversity effects	TBD	With increasing N addition rate, a larger decrease in complementarity effects compared to the increase of selection effects leads to a negative relationship between the net biodiversity effects and N addition rate	NA
H2: Overyielding change through time, which is opposite to the effects of N addition			
Year -> Complementarity effects	Positive	Complementarity effects increases with time due to belowground facilitation and faster cumulative N addition strengthening as experiment ages, especially under mixture community	Kahmen et al., (2006); Mason et al., (2020)
Year -> Selection effects	Negative	Selection effects decreases with time, or even reverse from positive in the early stage to negative in the later stage due to that a few established species growing well only in early stage and grow less competitive later	Fargione (2007)
Year -> Net biodiversity effects	Positive	The net biodiversity effects increase with time due to the contribution shift from selection effects to complementarity effects	Fargione et al., (2007); Marquard et al., (2009); Wagg et al., (2022)
H3: Overyielding change with increasing cumulative N addition			
Cumulative N addition -> Complementarity effects	Shift from negative to neutral	Complementarity effects decrease faster at low levels of cumulative N addition due to a potentially greater loss of positive interactions among species compared to high levels of cumulative N addition	NA
Cumulative N addition -> Selection effects	Positive	Selection effects stay positive at low levels of cumulative N addition and then increase faster with higher levels of cumulative N addition in the plot	NA

Cumulative N addition -> Net biodiversity effects	TBD	With increasing cumulative N addition, complementarity effects decrease faster at low levels of cumulative N addition and selection effects increases faster at higher levels of cumulative N addition, leading to a convex relationship between the net biodiversity effects and cumulative N addition.	NA

Table S3. Model settings and the result in summary table. Response variables include net biodiversity effect (NBE), complementarity effect (CE) and selection effect (SE). Explanatory variables include treatment (nitrogen addition, N), species richness (SR), N addition rate (N rate), cumulative N addition (CumuN). Asterisks (*) indicate statistical significance (ns, *P*>0.05; * *P*<0.05; ** *P*<0.01; ****P*<0.001).

Figure	Model	Response Variables	Terms	Estimate	Std.Error	df	t-value	p-value	Sig	R2m	R2c
2	~Treatment * SR + (1 + SR | Study)										
2a		NBE	(Intercept)	-57.777	70.7550	3.845	-0.8170	0.4617	ns	0.12	0.41
		NBE	N addition	16.173	28.1160	2384.488	0.575	0.5652	ns	0.12	0.41
		NBE	SR	105.286	37.8480	3.821	2.7820	0.0524	.	0.12	0.41
		NBE	N addition:SR	-11.196	9.1580	2384.478	-1.222	0.2217	ns	0.12	0.41
2b		CE	(Intercept)	-83.798	37.0110	1023.645	-2.2640	0.0238	*	0.07	0.20
		CE	N addition	72.904	50.9470	2388.231	1.431	0.15257	ns	0.07	0.20
		CE	SR	136.166	26.6670	3.229	5.1060	0.01211	*	0.07	0.20
		CE	N addition:SR	-54.169	16.5960	2388.226	-3.264	0.00111	**	0.07	0.20
2c		SE	(Intercept)	15.173	106.0280	4.189	0.1430	0.8928	ns	0.01	0.18
		SE	N addition	-36.943	56.4410	2387.518	-0.655	0.5128	ns	0.01	0.18
		SE	SR	-33.838	60.5700	4.026	-0.5590	0.606	ns	0.01	0.18
		SE	N addition:SR	39.41	18.3850	2387.396	2.144	0.0322	*	0.01	0.18
3	~ log(N rate+1) + (1 + log(N rate+1) | Study) - Include ambient										
3a		NBE	Intercept	293.777	48.3530	11.8540	6.0760	0.0001	***	0.00	0.40
		NBE	log(N rate+1)	-6.689	2.7250	44.325	-2.455	0.0181	*	0.00	0.40
3b		CE	Intercept	364.132	62.6080	7.9480	5.8160	0.0004	***	0.02	0.19
		CE	log(N rate+1)	-30.491	13.2770	7.603	-2.296	0.052398	.	0.02	0.19
3c		SE	Intercept	-38.412	76.2880	5.9510	-0.5040	0.6330	ns	0.00	0.13
		SE	log(N rate+1)	19.33	20.9230	6.18	0.924	0.39	ns	0.00	0.13
	~ log(N rate+1) + (1 + log(N rate+1) | Study) - Exclude ambient										
3d		NBE	Intercept	434.283	136.8190	7.1620	3.1740	0.0152	*	0.01	0.46
		NBE	log(N rate+1)	-37.525	24.9290	5.475	-1.505	0.1876	ns	0.01	0.46
3e		CE	Intercept	369.196	180.6780	9.3410	2.0430	0.0702	.	0.00	0.26
		CE	log(N rate+1)	-32.37	39.0900	8.635	-0.828	0.4299	ns	0.00	0.26
3f		SE	Intercept	-33.7	205.0600	83.5400	-0.1640	0.8700	ns	0.00	0.12
		SE	log(N rate+1)	15.32	53.7400	85.66	0.285	0.776	ns	0.00	0.12

4	~ log(CumuN+1) + I(log(CumuN+1)^2)+(1 + log(CumuN+1) | Study) [experimental addition]									
4a	NBE	Intercept	389.05	73.0930	11.3130	5.3230	0.0002	***	0.05	0.53
	NBE	log(CumuN+1)	-89.936	14.147	26.99	-6.357	8.31E-07	***	0.05	0.53
	NBE	I(log(CumuN+1)^2)	12.027	1.524	597.053	7.89	1.45E-14	***	0.05	0.53
4b	CE	Intercept	332.905	56.4900	10.4960	5.8930	0.0001	***	0.01	0.21
	CE	log(CumuN+1)	-43.23	12.601	599.566	-3.431	0.000644	***	0.01	0.21
	CE	I(log(CumuN+1)^2)	4.279	2.028	3334.599	2.109	0.034989	*	0.01	0.21
4c	SE	Intercept	-18.02	49.7850	3.5490	-0.3620	0.738	ns	0.01	0.13
	SE	log(CumuN+1)	-8.454	21.78	11.316	-0.388	0.705	ns	0.01	0.13
	SE	I(log(CumuN+1)^2)	4.238	2.93	113.153	1.447	0.151	ns	0.01	0.13
	~ log(CumuN+1) + I(log(CumuN+1)^2)+(1 + log(CumuN+1) | Study) [atmospheric deposition]									
4d	NBE	Intercept	318.97	74.7800	17.62	4.2660	0.0005	***	0.04	0.54
	NBE	log(CumuN+1)	-127.03	53.86	42.77	-2.358	0.022998	*	0.04	0.54
	NBE	I(log(CumuN+1)^2)	52.32	1.04E+01	704.29	5.033	6.15E-07	***	0.04	0.54
4e	CE	Intercept	390.97	133.8800	21.1700	2.9200	0.0081	**	0.00	0.24
	CE	log(CumuN+1)	-113.24	89.03	67.56	-1.272	0.20774	ns	0.00	0.24
	CE	I(log(CumuN+1)^2)	21.53	18.45	837.25	1.167	0.2436	ns	0.00	0.24
4f	SE	Intercept	-109.84	130.4100	27.7800	-0.8420	0.407	ns	0.03	0.20
	SE	log(CumuN+1)	13.01	98.89	105.01	0.132	0.896	ns	0.03	0.20
	SE	I(log(CumuN+1)^2)	30.84	22.1	576.32	1.396	0.163	ns	0.03	0.20
	~ log(CumuN+1) + I(log(CumuN+1)^2)+(1 + log(CumuN+1) | Study) [experimental addition + atmospheric deposition]									
4g	NBE	Intercept	249.016	52.6420	17.1850	4.7300	0.0002	***	0.00	0.41
	NBE	log(CumuN+1)	15.96	14.584	52.63	1.094	0.278809	ns	0.00	0.41
	NBE	I(log(CumuN+1)^2)	-2.403	1.652	365.216	-1.454	0.146673	ns	0.00	0.41
4h	CE	Intercept	262.165	72.7680	19.5020	3.6030	0.0018	**	0.02	0.19
	CE	log(CumuN+1)	36.406	26.876	78.522	1.355	0.17943	ns	0.02	0.19
	CE	I(log(CumuN+1)^2)	-8.206	2.92	783.896	-2.81	0.00508	**	0.02	0.19
4i	SE	Intercept	-16.677	93.0740	12.5480	-0.1790	0.8607	ns	0.01	0.14
	SE	log(CumuN+1)	-19.77	35.232	52.672	-0.561	0.5771	ns	0.01	0.14
	SE	I(log(CumuN+1)^2)	6.358	3.613	880.283	1.76	0.0788	.	0.01	0.14
S1	Treatment*log(Year) + (1 | SR) - BioCoN									
S1a	NBE	(Intercept)	18.255	34.8160	3.748	0.5240	0.6300	ns	0.11	0.17

	NBE	N addition	7.219	26.475	1404.017	0.273	0.785	ns	0.11	0.17
	NBE	log(Year)	85.3740	8.639	1396.8340	9.882	<2e-16	***	0.11	0.17
	NBE	N addition:log(Year)	-14.406	11.718	1404.0170	-1.229	0.219	ns	0.11	0.17
S1b	CE	(Intercept)	25.51	38.5700	12.65	-0.6610	0.5202	ns	0.08	0.10
	CE	N addition	104.34	43.78	1404.12	2.383	0.0173	*	0.08	0.10
	CE	log(Year)	131.3200	14.19	1220.6200	9.253	<2e-16	***	0.08	0.10
	CE	N addition:log(Year)	-92.43	19.38	1.40E+03	-4.77	2.03E-06	***	0.08	0.10
S1c	SE	(Intercept)	44.43	33.7300	1406	1.3170	0.1880	ns	0.02	0.02
	SE	N addition	-109.91	47.71	1406	-2.304	0.02138	*	0.02	0.02
	SE	log(Year)	-47.2700	14.93	1406.0000	-3.166	0.00158	**	0.02	0.02
	SE	N addition:log(Year)	86.56	21.12	1.41E+03	4.099	4.38E-05	***	0.02	0.02
S2	~ Treatment*Study, random= ~ 1|Year/Plot, correlation=corAR1(form = ~ 1|Year/Plot)									
S2a	NBE	Intercept	261.8099	43.0091	2662	6.0873	0.0000	***	0.18	0.91
	NBE	N addition	20.4067	44.7277	2662	0.4562	0.6483	ns	0.18	0.91
S2b	CE	Intercept	300.3024	70.0631	2662	4.2862	0.0000	***	0.09	0.89
	CE	N addition	-115.7748	79.2881	2662	-1.4602	0.1444	ns	0.09	0.89
S2c	SE	Intercept	-73.4749	74.7954	2662	-0.9823	0.3260	ns	0.06	0.89
	SE	N addition	181.5754	87.4883	2662	2.0754	0.0380	*	0.06	0.89

Table S4. Model selections for the effectiveness of cumulative N addition (CumuN). Response variables include net biodiversity effect (NBE), complementarity effect (CE) and selection effect (SE). Asterisks (*) indicate statistical significance (ns, P>0.05; * P<0.05; ** P<0.01; *** P<0.001).

**Response Variables**	**Model**	**Number of parameters**	**AIC**	**BIC**	**Negative log-likelihood**	**Deviance**	**Chisq**	**Df**	**Pr(>Chisq)**
NBE	~ log(N rate+1) * Year + (1 | SR)	6	49168	49205	-24578	49156			
NBE	~ log(N rate+1) * Year + log(CumuN+1) + (1 | SR)	7	49096	49140	-24541	49082	73.9080	1	2.20E-16 ***
CE	~ log(N rate+1) * Year + (1 | SR)	6	53063	53100	-26526	53051			
CE	~ log(N rate+1) * Year + log(CumuN+1) + (1 | SR)	7	53027	53071	-26507	53031	37.6870	1	8.31E-10 ***
SE	~ log(N rate+1) * Year + (1 | SR)	6	54528	54565	-27258	54516			
SE	~ log(N rate+1) * Year + log(CumuN+1) + (1 | SR)	7	54529	54572	-27257	54515	1.1655	1	2.80E-01 ns

Table S5. Model selections for cumulative N addition in first and second order (CumuN). Response variables include net biodiversity effect (NBE), complementarity effect (CE) and selection effect (SE). Asterisks (*) indicate statistical significance (ns, *P*>0.05; * *P*<0.05; ** *P*<0.01; *** *P*<0.001).

Response Variables	Model	Number of parameters	AIC	BIC	Negative log-likelihood	Deviance	Chisq	Df	Pr(>Chisq)	
NBE	~ log(CumuN + 1) + (1 + log(CumuN + 1) | Study)	6	48925	48962	-24456	48913				
NBE	~ log(CumuN + 1) + I(log(CumuN + 1)^2) + (1 + log(CumuN + 1) | Study)	7	48925	48968	-24455	48911	1.8454	1	0.1743	ns
CE	~ log(CumuN + 1) + (1 + log(CumuN + 1) | Study)	6	52180	52217	-26084	52168				
CE	~ log(CumuN + 1) + I(log(CumuN + 1)^2) + (1 + log(CumuN + 1) | Study)	7	52175	52219	-26081	52161	6.8441	1	0.0089	**
SE	~ log(CumuN + 1) + (1 + log(CumuN + 1) | Study)	6	52563	52600	-26276	52551				
SE	~ log(CumuN + 1) + I(log(CumuN + 1)^2) + (1 + log(CumuN + 1) | Study)	7	52561	52605	-26274	52547	4.0177	1	0.0450	*

Table S6. Model settings and the result in summary table for the effects of evenness change with cumulative nitrogen addition and time. Response variables include net biodiversity effect (NBE), complementarity effect (CE) and selection effect (SE). Explanatory variables include treatment (N addition), species richness (SR), Year, cumulative N addition (CumuN). Asterisks (*) indicate statistical significance (ns, *P*>0.05; * *P*<0.05; ** *P*<0.01; ****P*<0.001).

	Model	Response Variables	Terms	Estimate	Std.Error	df	t-value	p-value	Sig	R2m	R2c
Cumulative N addition	~ evenness + log(CumuN+1) + I(log(CumuN+1)^2)+(1 + log(CumuN+1) | Study)										
		NBE	(Intercept)	449.73	60.5	15.03	7.43	0	***	0.05	0.47
		NBE	evenness	-324.4	9.54	24861.7	-34.01	<2e-16	***	0.05	0.47
		NBE	log(CumuN+1)	5.74	9.14	24.9	0.63	0.536	ns	0.05	0.47
		NBE	I(log(CumuN+1)^2)	-2.27	0.59	17541.44	-3.82	0.0001	***	0.05	0.47
		CE	Intercept	219.2	76.93	14.35	2.85	0.0126	*	0.02	0.25
		CE	evenness	-18.86	16.38	24405.55	-1.15	0.2496	ns	0.02	0.25
		CE	log(CumuN+1)	65.41	18.05	20.97	3.62	0.0016	**	0.02	0.25
		CE	I(log(CumuN+1)^2)	-11.66	1.02	19215.6	-11.41	<2e-16	***	0.02	0.25
		SE	Intercept	290.67	94.48	15.3	3.08	0.0075	**	0.04	0.27
		SE	evenness	-439.55	17.7	24204.45	-24.83	<2e-16	***	0.04	0.27
		SE	log(CumuN+1)	-61.16	24.03	17.86	-2.55	0.0204	*	0.04	0.27
		SE	I(log(CumuN+1)^2)	10.19	1.11	21722.96	9.21	<2e-16	***	0.04	0.27
Year effect on BioCoN experiment	~ evenness + Treatment*log(Year) + (1 | SR) - BioCoN										
		NBE	(Intercept)	51.83	28.04	2.49	8.98	0.0059	**	0.23	0.27
		NBE	evenness	-760.36	22.79	13953.30	-33.37	<2e-16	***	0.23	0.27
		NBE	N addition	-35.52	8.35	15348.70	-4.25	<2e-16	***	0.23	0.27
		NBE	log(Year)	67.29	2.68	15348.91	25.09	<2e-16	***	0.23	0.27
		NBE	N addition:log(Year)	-3.70	3.56	15348.09	-1.04	0.2984	ns	0.23	0.27
		CE	(Intercept)	-41.73	29.59	3.42	-1.41	0.2427		0.10	0.12
		CE	evenness	62.43	35.58	9261.67	1.75	0.0794	.	0.10	0.12
		CE	N addition	87.61	13.08	15349.48	6.70	0.0000	***	0.10	0.12

	CE	log(Year)	134.15	4.20	15349.81	31.94	<2e-16	***	0.10	0.12
	CE	N addition:log(Year)	-89.16	5.57	15348.26	-16.01	<2e-16	***	0.10	0.12
	SE	(Intercept)	331.75	43.28	2.53	7.67	0.0082	**	0.08	0.13
	SE	evenness	-931.51	37.13	13585.24	-25.09	<2e-16	***	0.08	0.13
	SE	N addition	-136.54	13.61	15348.76	-10.03	<2e-16	***	0.08	0.13
	SE	log(Year)	-75.16	4.37	15349.00	-17.19	<2e-16	***	0.08	0.13
	SE	N addition:log(Year)	93.56	5.80	15348.08	16.14	<2e-16	***	0.08	0.13

Supplementary References

1. Isbell, F. *et al.* Nutrient enrichment, biodiversity loss, and consequent declines in ecosystem productivity. *Proc. Natl. Acad. Sci. U. S. A.* **110**, 11911–11916 (2013).
2. Reich, P. B. et al. Plant diversity enhances ecosystem responses to elevated CO2 and nitrogen deposition. *Nature* **410**, 809–812 (2001).
3. Lanta, V. & Lepš, J. Effects of species and functional group richness on production in two fertility environments: an experiment with communities of perennial plants. *Acta Oecologica* **32**, 93–103 (2007).
4. Mason, N. W. H. et al. Resource-use efficiency drives overyielding via enhanced complementarity. *Oecologia* **193**, 995–1010 (2020).
5. Pichon, N. A. et al. Decomposition disentangled: A test of the multiple mechanisms by which nitrogen enrichment alters litter decomposition. *Functional Ecology* **34**, 1485–1496 (2020).
6. Pontes, L. da S., Maire, V., Louault, F., Soussana, J. F. & Carrère, P. Impacts of species interactions on grass community productivity under contrasting management regimes. *Oecologia* **168**, 761–771 (2012).
7. Roscher, C., Schmid, B., Kolle, O. & Schulze, E. D. Complementarity among four highly productive grassland species depends on resource availability. *Oecologia* **181**, 571–582 (2016).
8. Kirwan, L. The Agrodiversity Experiment three years of data from a multisite study in intensively managed grasslands. *Ecology* **95**, 2680 (2014).
